# The Cervical Lymphatics in Phthisis

**Published:** 1909-01-16

**Authors:** 


### Medicine.

### THE CERVICAL LYMPHATICS IN PHTHISIS.

Among students of tuberculosis the consensus of opinion in the present day seems to be that the tubercle bacillus gains entrance into the body through the lymphatic system, and that tuberculosis is primarily a lymphatic disease. This has not been settled absolutely, but it can be accepted as a working hypothesis. The work of a host of observers seems to prove conclusively that the lymphatic system is the ordinary channel of entrance of the tubercle bacillus, and that the lymphatic glands are primarily involved in nearly all cases, although they seldom give rise to symptoms of sufficient importance to attract attention. The glands which are usually affected first, if not the cervical, are either the bronchial or the mesenteric groups.

Whether the respiratory tract or the alimentary canal is the more frequent port of entry of the tubercle bacillus has not been settled, but investigators agree that entrance may take place by either or even by both. For a while it was held that admission was always by the respiratory tract; then the other extreme view, that it was always by the alimentary canal, was adopted. Thorough and painstaking work by advocates of both theories has finally proved that admission is by both routes, and perhaps as often by the one as by the other. Entrance often occurs by way of the tonsils and the pharynx, but these parts are looked upon as a subdivision of the alimentary canal.

It has been fairly well proved that the tubercle bacillus may pass through a lymphatic gland without leaving behind any traces of its presence. In such cases no visible damage is done to the lymphatic gland. Often, however, a histological study of a gland which has been invaded reveals changes indicative of bacillary invasion. Inoculation of animals with the gland itself often reveals the presence of tubercle bacilli, though they are not to be discovered microscopically in the gland itself. It is possible, as Raw suggests, that there is a difference in the way in which bovine and human tubercle bacilli behave, and that whereas the former produce serious lesions and caseation, the latter are able to pass through the lymphatic glands without doing much visible damage.

A lymphatic infection is naturally propagated viâ the lymphatic system, passing readily through the vessels but becoming retarded at each lymph gland until the functional activity of that node has been overcome. Much has been written lately in regard to the relationship between tonsillitis or tonsillar affections and disease of the lung and pleura. It might be supposed that tubercle bacilli, gaining entrance by the tonsils, might traverse the cervical lymphatic channels, reach the supra-clavicular glands, and thence directly infect the pleura and lung. This would afford a very pretty explanation of the fact that the common site for tubercle of the lung to begin at is the apex, or rather a spot about an inch below the actual apex. This theory, however, seems to be negatived by experimental work, such as that of Fleiner, Beitzke, and G. B. Wood, which seems to indicate that there is no anatomical connection between the lower cervical lymphatic glands and the lymphatic vessels of the pleura, nor even any direct continuity between an enlarged supra-clavicular gland and the corresponding apical pleura. Wood seems to incline to the view that microbial infection viâ the tonsil certainly can produce pleurisy, but that it does so not by a direct spread, but indirectly viâ the blood stream. Whether this be so or not requires a great deal more combined experimental and clinical work to decide; but the question of whether or not tubercle bacilli absorbed by the tonsils can and do commonly cause pleurisy and phthisis, and if so, then how these bacilli reach the lung from the tonsils, is one of such extreme importance that we feel justified in quoting Wood's* experiments at some length, and giving his views upon the arrangement of the peritonsillar and cervical lymphatic vessels and glands. He says: "Concerning lymphatic infection of the pleura from the throat; it is essential first to find out whether there normally exist lymphatics connecting the upper deep cervical lymph glands with the apical parietal pleura; secondly, if such do not exist, whether diseased processes, by cutting off the normal flow of lymph, can produce a collateral circulation whereby lymph from the upper glands of the neck may come in contact with the pleura or its regionary glands."

The lymph-glands of the neck are divided into two main groups, the superficial and the deep. The superficial glands drain into the deep ones, generally into the gland which is anatomically the nearest neighbour.

The deep glands situated on each side of the neck comprise from 15 to 30 nodes on an average, with wide variations above and below this. They have been variously subdivided for purposes of description, although anatomically they seem to make up one continuous mass. They extend from just beneath the ear downward under the sterno-cleido-mastoid muscle, sometimes only as far as the point where the omo-hyoid crosses the vessels and nerves, but occasionally reaching as far as the junction of the internal jugular and subclavian veins. A gland situated in the notch formed by these veins is, as Wood insists, of the utmost importance.

The more superficial division of the deep lateral chain lies posteriorly, and is called the external group. The external glands are generally small, and placed in part beneath the posterior border of the sterno-mastoid; occasionally they extend so far down the anterior border of the trapezius muscle as to come into rather close relation with the supra-clavicular glands. The anterior or deeper division of the main group is placed directly over the great vessels of the neck, and is termed the internal jugular group. It is situated beneath the anterior border of the sterno-mastoid muscle, and when enlarged its glands may be forced anteriorly until some of them appear immediately below the angle of the jaw.

* Report of Henry Phipps Institute.

The external glands empty their lymph into the internal jugular group, which in their turn drain one into the other as they descend the neck, finally emptying into the jugular lymph trunk. This lymph trunk empties into the junction of the internal jugular and subclavian veins, and on the left side of the neck is occasionally joined by the thoracic duct just before it enters the vein.

The supra-clavicular glands occupy the sub-clavian triangle, and the possibility of connection between this group and the deep cervical glands of the neck is the chief contention in the question of infection from the throat reaching the pleura. G. B. Wood, whom we are quoting, argues against such direct connection, though we are not sure but that his experiments might not bear an exactly reverse interpretation. The point is not settled, but it is one that is so important that all should be familiar with the work that is being done upon it, even though the results so far seem indecisive. The supra-clavicular glands receive lymphatics from the posterior part of the hairy scalp and from the muscles of the neck; also from the skin of the arm and chest, and an efferent branch from the humeral chain cf the axillary group. Their efferent duct generally empties into the jugular trunk just before it enters the vein.

Wood suggests that the injection of the supra-clavicular gland in one of his cases took place against the normal direction of current, owing to the node situated in the venous notch obstructing the downward flow, so that the fluid on entering this node passed as readily up the efferent of the supra-clavicular gland as into the jugular trunk. It is by no means clear, however, what right one has to make any such assumption, rather than to allow that materials may naturally pass from the upper cervical lymphatics directly into the supra-clavicular glands, and thus into close relationship with the pleura. It is true that the supra-clavicular glands are themselves a little distance from the apical pleura, but they are connected with the node occupying the venous notch, and this is separated from the pleura only by the carotid artery and its sheath. Enlargement of this node sufficient to cause displacement either anteriorly or posteriorly would bring its capsule into almost direct contact with the pleura.

It is interesting to note the condition of the supra-clavicular glands as found at autopsies upon phthisical patients. Out of seventeen consecutive cases tuberculous foci were found microscopically in these glands in fifteen. This is a very high percentage, and it makes one wonder whether the tubercle bacilli got into the glands after the lungs had first been affected, or whether the organisms had got into the glands first and thence had infected the lung. It is quite true that patients who suffer from extensive tuberculosis of the cervical glands in early life seem particularly immune to phthisis; it is also quite true that there was no obvious affection of the supra-clavicular glands during life in the above fifteen cases in which tubercles were found in them microscopically after death. It may be, therefore, that when the glands catch up the bacilli and become obviously caseous, extension is being arrested, and that there is another type of tuberculous lymphadenitis in which the organisms are allowed to extend along the lymphatic chain without causing clinical signs of any lymphatic infection at all. It is a remarkable fact that the supra-clavicular lymphatic glands exhibit tuberculous foci in such a large number of phthisical cases. The subject of the path of infection in phthisis is one upon which views that seemed absolutely stable a few years ago are now looked upon as very far from certain. The view that phthisis is due to tubercle bacilli reaching the lungs viâ the bronchioles is being boldly challenged. Lymphatic infection from the upper respiratory channels, and thence an extension to the lung from the supra-clavicular or closely related lymph nodes would explain very prettily why phthisis always begins near the apex of the lung. The subject is one upon which a great deal of research work could comparatively easily be done. An accumulation of evidence upon the microscopical characters of the different groups of lymphatic glands in the neck, thorax, and abdomen in fatal cases of phthisis might lead to very important discoveries.

